# Small cell carcinoma of the appendix

**DOI:** 10.1186/1477-7819-6-4

**Published:** 2008-01-15

**Authors:** Anna M O'Kane, Mark E O'Donnell, Rajeev Shah, Declan P Carey, Jack Lee

**Affiliations:** 1 Department of Surgery, Belfast City Hospital, Lisburn Road, Belfast BT9 7AB. Northern Ireland, UK; 2 Department of Histopathology, Belfast City Hospital, Lisburn Road, Belfast BT9 7AB. Northern Ireland, UK

## Abstract

**Background:**

An extrapulmonary small cell carcinoma is a rare condition. It has similar histological features to pulmonary small cell carcinoma and is equally aggressive.

**Case presentation:**

We present the case of a 60-year-old woman who presented with right upper quadrant pain. Computerised tomography revealed an appendiceal lesion and multiple liver metastases. Exploratory laparotomy and right hemicolectomy was performed with histopathological analysis confirming a primary small cell carcinoma of her appendix.

**Conclusion:**

This is the first reported case of a pure extrapulmonary carcinoma arising from the appendix.

## Background

Extrapulmonary small cell carcinomas (ESC) are rare. Many different sites of origin have been described including kidney, bladder, prostate, endometrium, salivary glands, nasal sinuses and intestinal tract [1-5]. Primary colonic ESC remains the rarest and most aggressive. There is an equal sex distribution with a preponderance for middle aged patients. We present a case of a 60-year old female with a primary small cell carcinoma of the appendix with liver metastasis.

## Case presentation

A 60-year-old female was admitted with a 4-day history of right upper quadrant pain. She was treated with oral antibiotics for suspected acute cholecystitis. She had a past medical history of Type-2 diabetes and hypertension. She was a non-smoker. The patient had no fever, sweating or rigors but described similar intermittent pain with associated nausea and vomiting over the preceding 6-weeks. On examination, the patient was comfortable and well nourished. Her clinical parameters (pulse and blood pressure) were normal and she was apyrexic. Abdominal examination revealed right upper quadrant tenderness with a palpable liver edge. There were no other masses or organomegaly.

**Figure 1:**
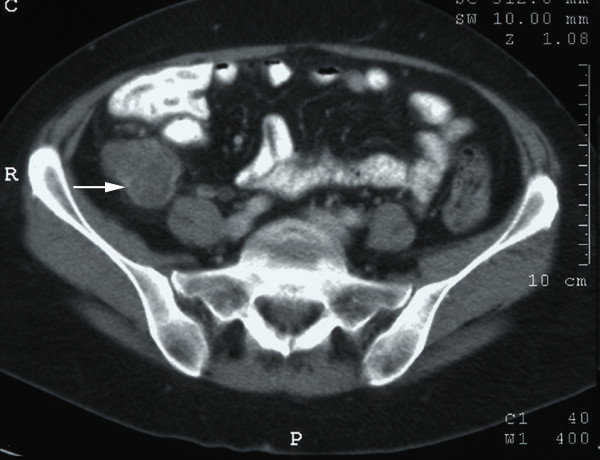
Contrast-enhanced computerised tomography scan of the abdomen demonstrating a 6 cm × 7 cm tumour mass in the right iliac fossa (white arrow). Although the tumour mass was inseparable from the lower pole of the caecum, it appeared separate from the ileo-caecal valve.

**Figure 2:**
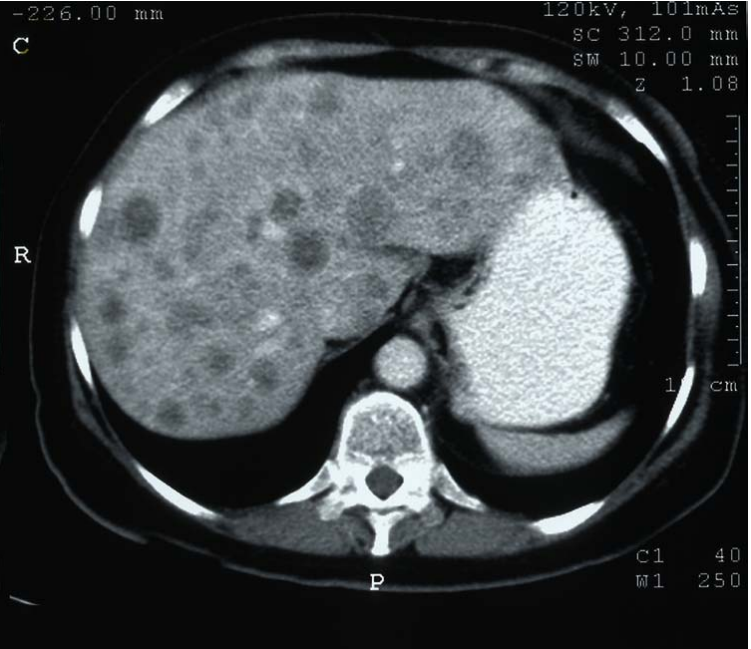
Contrast-enhanced computerised tomography scan of the abdomen demonstrating multiple liver metastases.

Haematological analyses showed a haemoglobin level of 13.9 g/dl, white cell count 10.8 × 10^9^/l and C-reactive protein 19 mg/L. All other indices were normal as were the plain chest and abdominal X-rays. An abdominal ultrasound showed a markedly abnormal liver appearance with multiple hypoechoic lesions suggestive of multiple metastases. The remainder of the biliary tree was normal. A contrast-enhanced computerised tomography (CT) scan of the chest, abdomen and pelvis confirmed multiple liver metastases within both lobes of the liver but also a 6 × 7 cm tumour mass in the right iliac fossa (Figures 1 &2). There was associated lymphadenopathy extending through the ileo-colic branch of the superior mesenteric artery and further large lymph nodes measuring up to 1.9 cms in diameter in the aorto-caval and para-aortic regions. Although the lesion was separate from the ileo-caecal valve, radiological imaging suggested an appendiceal or caecal origin. Further extrinsic pressure to the distal third of the right ureter was present with mild hydronephrosis. No lung parenchymal abnormality was identified.

Gastrointestinal investigation with colonoscopy was planned but cancelled due to deteriorating symptomatology with conservative treatment. Laparotomy revealed a large tumour mass which appeared to originate from the ascending colon. This was adherent to but not invading the right ureter and lateral abdominal wall. Liver metastases and multiple enlarged lymph nodes along the ileo-colic branch of the superior mesenteric artery were also identified. Due to the involvement of surrounding structures and a suspected caecal origin a right hemicolectomy was performed with a primary ileo-colic anastomosis. The right ureter was preserved as the tumour was dissected free of both the ureter and lateral abdominal wall. No synchronous colorectal tumour was identified during surgery.

**Figure 3:**
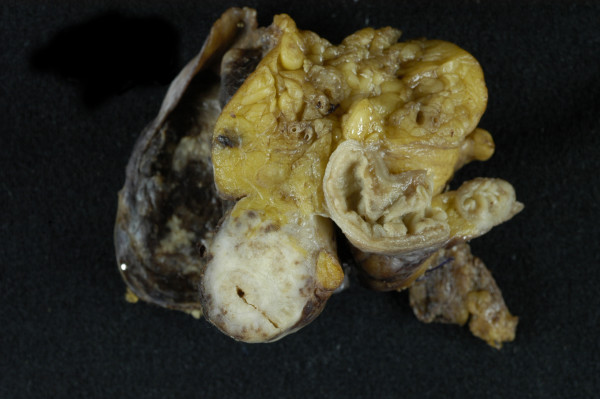
Macroscopic image demonstrating the extrapulmonary small cell carcinoma which had surrounded and replaced the appendix without caecal involvement.

**Figure 4:**
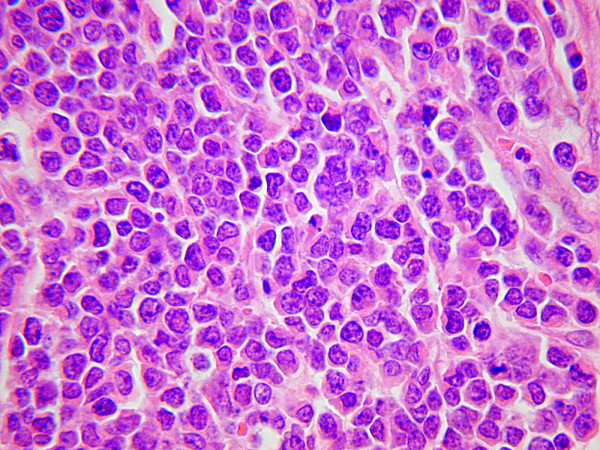
High-power microscopic view of the small cell carcinoma showing hyperchromatic nuclei, nuclear moulding and clumping of the nuclear chromatin.

Macroscopic examination showed that the tumour had replaced the appendix without caecal involvement (Figure 3). Histological examination showed a small cell carcinoma tumour composed of small cells with round to ovoid nuclei, dispersed chromatin, scanty cytoplasm and abundant mitoses (Figure 4). The tumour had extended through the peritoneum and involved the surrounding adipose tissue replacing the entire appendiceal mucosa. There was extensive lymphovascular invasion and metastatic involvement of regional lymph nodes. Immunohistochemistry demonstrated positivity for the epithelial markers CAM 5.2 and AE1/AE3 and the neuroendocrine markers PGP 9.5, synaptophysin and TTF1. Ki-67 staining index was approximately 90%. Tumours cells were negative for cytokeratin 7, cytokeratin 20, CD 45 (LCA), desmin, WT-1, CD 56, chromogranin and CD 99. The morphology and immunohistochemical features were in keeping with a neuroendocrine carcinoma of small cell type. In the absence of an identified pulmonary tumour, a diagnosis of primary appendiceal small cell carcinoma was made.

She made an uneventful surgical recovery and was transferred to the oncology department 12-days after surgery for palliative chemotherapy. The patient developed a right flank abscess after receiving one cycle of carboplatin. The abscess was drained percutaneously. Subsequently the patient was referred to the palliative care team and passed away 2-months after surgery. A post mortem was not performed.

## Discussion

Undifferentiated small cell carcinoma (SCC) is an aggressive lung tumour accounting for 15% of all lung cancers [1]. Extrapulmonary small cell carcinomas (ESC) in comparison are rare with an incidence between 0.1–0.4% of all cancers [2]. Approximately 2.5% of all SCC's arise in extrapulmonary sites such as the salivary glands, pharynx, larynx, nasal sinuses, pancreas, oesophagus, colon, rectum, skin and cervix [2-5]. Colorectal ESCs are rare with an incidence of 0.3% of all colorectal cancers and like SCC of the lung, are aggressive malignancies with early metastasis and have an overall 5-year survival of 13% [6]. Kim *et al *(2004) reported a 12.5% incidence of colorectal ESC with 3 patients affected from a retrospective review of 24 patients with ESC [7].

Age and sex distribution for ESC are similar to that seen in adenocarcinoma of the colon [6]. Although smoking is clearly implicated in the formation of pulmonary SCC, its association with ESC is not clearly documented. This patient was a non-smoker but there was a family history of lung cancer with an elderly brother who died in his fifties. The type of lung cancer affecting the patient's brother was not determined and therefore it is unclear whether her family history of lung cancer had a causative role either.

SCC is thought to originate from neuroendocrine cells, which are found in the epithelium of many mucosal surfaces including the gastrointestinal tract [6]. Despite evidence of neuroendocrine involvement, the origin of ESC is still unclear as development from undifferentiated airway epithelium has also been suggested along with the amine precursor uptake and decarboxylation (APUD) system hypothesis which proposes a common ancestral cell derived from the neural crest, which then migrates to various epithelial tissues and sites within the body [8,9].

Histopathological diagnosis can be confirmed by the classic appearance of small round to oval shaped cells with a finely granular and hyperchromatic nucleus, inconspicuous nucleoli and scanty cytoplasm on light microscopy [8]. SCC's show a strong and diffuse immunoreactivity for CD 56 and 80% positivity for TTF-1 tumour markers [10,11]. TTF-1 is positive in most cases of pulmonary small cell carcinoma, but also shows positive staining with many high-grade neuroendocrine carcinomas of non-pulmonary origin. The importance of TTF-1 is to exclude metastatic Merkel cell carcinoma, which is TTF-1 negative [11]. Due to the extent of disease in our case it was not possible to assess dysplastic changes of the surrounding mucosa. In the absence of a lung primary combined with the immunohistochemical profile of the appendiceal tumour suggests that this patient had a pure extrapulmonary SCC of her appendix. Although carcinoid tumours account for 32–35% of all appendiceal neoplasms, SCC's of the appendix are rarer with only one previously reported case by Rossi *et al *and this was mixed with adenocarcinoma [12-14]. To the authors' knowledge this is the first reported case of a pure small cell carcinoma of the appendix. Further investigative modalities with CT imaging and bronchoscopy are mandatory to exclude a pulmonary origin [2]. Although this patient had a positive family of pulmonary neoplasia, she was a non-smoker with no respiratory symptomatology and had a normal chest CT scan. Following consultation with the respiratory department following surgery, no further investigation was requested as oncological treatment was the priority.

Unfortunately clinical presentation of ESC carcinoma is usually at an advanced stage due to the aggressive nature of the disease. Therapeutic modalities are determined by the location and extent of disease. Chemotherapy remains the treatment of choice. The role of radiotherapy and surgical intervention remain limited, with surgery often only being used for the treatment of localised disease [15]. Combination chemotherapy regimens using cisplatin-etoposide are the most commonly used with response rates of up to 70% [4]. There are no definite chemotherapeutic regimens for ESC of the colon due to the small patient numbers and clinically advanced disease at presentation.

The prognosis for ESC is similar to pulmonary SCC's and remains poor with a rapidly deteriorating clinical course. Five-year survival is less than 13% [15]. The mean survival for gastrointestinal ESC is less than 5-months with a 3- and 8-month mean survival for extensive and localised disease respectively [16].

## Competing interests

The author(s) declare that they have no competing interests.

## Authors' contributions

AOK: Involved in the literature review, manuscript preparation and manuscript editing. MEOD: Involved in the conception of the report, literature review, manuscript preparation, manuscript editing and manuscript submission. RS: Involved in the critical analysis of the histopathology in the case report and manuscript review. PDC: Involved in the manuscript editing and manuscript review. JL: Involved in manuscript editing and manuscript review.

All authors have read and approved the final manuscript.
